# A loss of host-derived MMP-7 promotes myeloma growth and osteolytic bone disease in vivo

**DOI:** 10.1186/s12943-017-0616-9

**Published:** 2017-02-28

**Authors:** S. T. Lwin, J. A. Fowler, M. T. Drake, J. R. Edwards, C. C. Lynch, C. M. Edwards

**Affiliations:** 1 Nuffield Department of Surgical Sciences, University of Oxford, Oxford, UK; 2 Nuffield Department of Orthopaedics, Rheumatology and Musculoskeletal Sciences, Botnar Research Centre, University of Oxford, Old Road, Oxford, OX3 7LD UK; 3 Department of Medicine/Clinical Pharmacology, Vanderbilt Center for Bone Biology, Nashville, USA; 4 Division of Endocrinology, Mayo Clinic College of Medicine, Rochester, MN USA; 5 Department of Tumor Biology, H. Lee Moffitt Cancer Center, Tampa, FL USA

**Keywords:** Multiple myeloma, Bone, MMP-7, Osteoclast, Microenvironment, Bone disease, Mouse model

## Abstract

Matrix metalloproteinases (MMPs) play a critical role in cancer pathogenesis, including tumor growth and osteolysis within the bone marrow microenvironment. However, the anti-tumor effects of MMPs are poorly understood, yet have significant implications for the therapeutic potential of targeting MMPs. Host derived MMP-7 has previously been shown to support the growth of bone metastatic breast and prostate cancer. In contrast and underscoring the complexity of MMP biology, here we identified a tumor-suppressive role for host MMP-7 in the progression of multiple myeloma in vivo. An increase in tumor burden and osteolytic bone disease was observed in myeloma-bearing MMP-7 deficient mice, as compared to wild-type controls. We observed that systemic MMP-7 activity was reduced in tumor-bearing mice and, in patients with multiple myeloma this reduced activity was concomitant with increased levels of the endogenous MMP inhibitor, tissue inhibitor of metalloproteinases-1 (TIMP-1). Our studies have identified an unexpected tumour-suppressive role for host-derived MMP-7 in myeloma bone disease in vivo, and highlight the importance of elucidating the effect of individual MMPs in a disease-specific context.

## Background

Matrix metalloproteinases (MMPs) are key regulators of tumor-host interactions due to their ability to alter the activity of multiple substrates, including growth factors and cytokines [1]. MMPs have been implicated in the pathogenesis of many tumors, and increasing evidence suggests a critical role for host-derived MMPs in cancer progression [2–6]. As such, MMPs represent an attractive therapeutic target, although to date, clinical trials of MMP inhibitors have largely been unsuccessful [7]. One explanation for such failure is likely the broad-spectrum nature of the inhibitors, leading to a multitude of off target effects. Therefore, it is necessary to elucidate the specific mechanisms of individual MMPs in a disease specific context, in order to develop the most effective therapeutic strategy.

The bone marrow provides a specialized microenvironment for the development of solid tumor metastases including breast and prostate cancer, and hematological malignancies such as multiple myeloma. The reciprocal relationship between tumor burden and osteolytic bone disease results in rapid increases in tumor burden and bone loss. Resultant tumors are generally incurable and treatments largely palliative [8]. In order to identify new therapeutic targets and approaches, it is necessary to elucidate the complex mechanisms that control tumor growth and survival within the bone marrow microenvironment. Interactions between tumor cells and cells of the host bone marrow microenvironment are critical in both tumor growth and survival, and the development of the associated bone disease [9]. MMPs have been implicated in these interactions, in particular MMP-7, which has previously been shown to be highly expressed within the tumor-bone microenvironment and to promote breast and prostate cancer osteolysis in vivo [2, 3]. In multiple myeloma, a number of MMPs have been implicated in disease progression, most notably MMP-2 and MMP-9 [10–17]. Indeed, host-derived MMP-9 has been shown to promote disease pathogenesis in a murine model of myeloma in vivo [18]. In vitro studies suggest that myeloma-derived MMP-7 can activate stromal MMP-2 [17], however the in vivo role of MMP-7 in myeloma pathogenesis thus far is unknown.

While selective pharmacological inhibition of MMPs remains a challenge, understanding the biology through which they mediate their effects can result in the identification of novel targetable substrates/pathways. In the present study, we identified an unexpected tumor-suppressive role for MMP-7 in myeloma pathogenesis. By combining a murine model of myeloma with MMP-7 deficient mice, our results suggest that, in contrast to breast and prostate cancer bone metastasis, a loss of host-derived MMP-7 increases tumor growth and osteolytic bone disease. Analysis of patients with multiple myeloma revealed a decrease in systemic MMP-7 activity in multiple myeloma in vivo. Taken together, our results identify a novel anti-tumor role for MMP-7 in vivo and highlight the importance of elucidating the function of individual MMPs in a disease-specific context.

## Materials and methods

### Cell culture and stable cell lines

The 5TGM1-GFP myeloma cell line was cultured as previously described [19]. 2T3 osteoblasts were cultured in DMEM supplemented with 10% FCS and L-glutamine. All cell lines were confirmed as mycoplasma-free. 2T3 osteoblasts were transduced with 10^5^ IFU/ml of lentiviral particles from pLenti-suCMV (mMMP-7)-Rsv (Puro) MMP-7 or null control lentivector (Amsbio). MMP-7 overexpressed and null transduced cells were cultured in media as described above and supplemented with 5 μg/ml puromycin. Overexpression of MMP-7 was confirmed by real-time PCR, and cells were continuously cultured in puromycin to maintain MMP-7 overexpression.

### Animal models

Animal studies were approved by the Vanderbilt University Institution of Animal Care and Use Committee. Double null recombinase-activating gene-2 (RAG-2) and MMP-7 mice were generated as previously described [2]. 8 week old female Rag2^−/−^;MMP-7^−/−^ (MMP-7^−/−^) or wild-type littermate controls (Rag2^−/−^;MMP-7^+/+^) were intravenously inoculated with either 10^6^ 5TGM1-GFP cells or vehicle control (PBS). Sera were assayed for monoclonal mouse IgG_2bκ_ paraprotein as described previously [19]. Tumor burden in bone marrow and spleen was quantitated by flow cytometric analysis of the proportion of GFP-positive myeloma cells [20]. Myeloma bone disease was assessed by microCT analysis of trabecular bone volume and number of osteolytic lesions, as described previously [20]. Apoptosis and proliferation were assessed by TUNEL staining and phospho-histone H3 staining respectively, using immunohistochemistry techniques described previously [21].

### Patient samples

Patient samples were obtained with approval from the Institutional Review Board and Biospecimen Protocol Review Group of the Mayo Clinic College of Medicine. Serum samples from patients with multiple myeloma, and the respective age- and sex-matched controls, were obtained through collaboration with M.T.D.

### MMP activity

MMP-7 activity in serum was measured using human or mouse specific MMP-7 activity assays (Quickzyme Biosciences, Leiden, The Netherlands), according to the manufacturer’s instructions.

### RT-PCR

RNA from cell lines was isolated using the RNeasy kit (Qiagen). cDNA was generated using the iScript cDNA Synthesis Kit (BioRad). MMP-7, TIMP-1 and GAPDH gene expression was quantitated by PCR.

### Cell viability

5TGM1-GFP or RPMI 8226 myeloma cells were plated at 5 × 10^5^ cells/ml and treated with 0–1000 ng/ml recombinant human active MMP-7 in serum-free media (Millipore). Proliferation was measured using a colorimetric CellTiter 96® AQueous Non-Radioactive Cell Proliferation Assay (Promega).

### ELISAs and immunoblotting

Serum concentrations of MMP-7 and TIMP1 were measured according to the manufacturers’ instructions using commercially available ELISA kits (R&D Systems). 5TGM1-GFP myeloma cells were treated with 100 ng recombinant mouse galectin-3 (R&D Systems) in the presence and absence of 10–100 ng MMP-7 and cleavage fragments detected by silver staining

### Statistics

Statistical significance was determined using a Mann-Whitney *U* Test or one-way ANOVA and Tukey-Kramer posthoc test and considered significant for *p* < 0.05. In vivo experiments were repeated on a minimum of two separate occasions and in vitro experiments were performed a minimum of three separate occasions. Data are represented as mean ± SE unless otherwise stated.

## Results

**Fig. 1:**
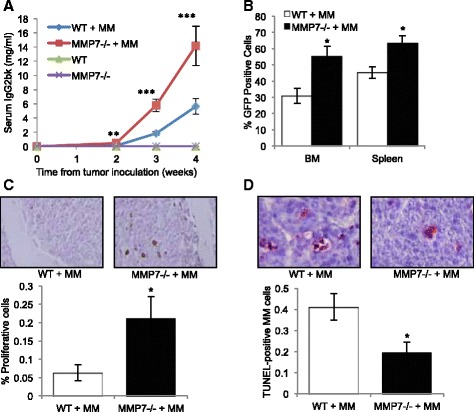
Myeloma tumor burden is increased in MMP-7-deficient mice. Wild-type (WT) or MMP-7 homozygous deficient (MMP-7^−/−^) mice were inoculated with 5TGM1 myeloma cells (MM) or vehicle control. Tumor burden was measured by (**a**) serum IgG2bκ concentrations and (**b**) flow cytometric analysis of GFP-positive myeloma cells in the bone marrow and spleen. **c** Myeloma cell proliferation in the bone marrow was quantitated by phospho-histone H3 staining, (**d**) Myeloma cell apoptosis was quantitated by TUNEL staining. Data represent mean ± SEM. Mann Whitney *U* test *p < 0.05, **p < 0.01, ***p < 0.001 as compared to WT + MM. (WT, *n* = 7; MMP-7^−/−^, *n* = 4; WT + MM, *n* = 8; MMP-7^−/−^ + MM, *n* = 5)

**Fig. 2:**
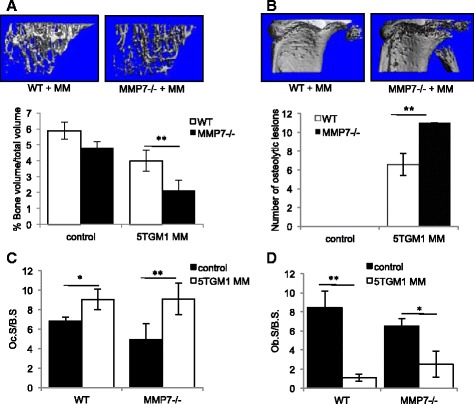
Osteolytic bone disease is increased in myeloma-bearing MMP-7-deficient mice. Wild-type (WT) or MMP-7 homozygous deficient (MMP-7^−/−^) mice were inoculated with 5TGM1 myeloma cells (MM) or vehicle control. MicroCT analysis demonstrated that trabecular bone volume was decreased (**a**) and osteolytic lesions were increased (**b**) in myeloma bearing MMP-7^−/−^ mice, as compared to myeloma bearing WT mice. Histomorphometric analysis demonstrated an increase in osteoclasts (**c**) and a decrease in osteoblasts (**d**) in myeloma-bearing WT and MMP-7−/− mice as compared to control. Data represent mean ± SEM. Mann Whitney *U* test *p < 0.05, **p < 0.01, ***p < 0.001 (WT, *n* = 7; MMP-7^−/−^, *n* = 4; WT + MM, *n* = 8; MMP-7^−/−^ + MM, *n* = 5)

Previously, host derived MMP-7 has been shown to promote the growth of bone metastatic breast and prostate cancer. To determine the role of host-derived MMP-7 in myeloma pathogenesis, we used immunocompromized (RAG-2^−/−^) mice that were wild type or null for MMP-7 and can be successfully engrafted with the 5T myeloma model [18]. The 5T model is well-characterized and closely mimics human myeloma, however 5T myeloma cells will only grow in syngeneic C57BL/KaLwRij mice, bg/nu/Xid mice or Rag2^−/−^ mice [18]. Littermate wild-type (WT, Rag2^−/−;^MMP-7^+/+^) and MMP-7 homozygous deficient (MMP-7^−/−^, Rag2^−/−^; MMP-7^−/−^) mice were inoculated with 5TGM1 myeloma cells. A significant increase in tumor burden in myeloma-bearing MMP-7^−/−^ mice was demonstrated by an increase in myeloma-specific IgG_2bκ_ serum concentrations and GFP-positive myeloma cells in the bone marrow and spleen, as compared to myeloma-bearing WT mice (Fig. 1a and b). Subsequent histological assessment of the bone marrow demonstrated that the proliferative rate of myeloma cells within the MMP-7^−/−^ environment was greater than that in WT animals (Fig. 1c). A significant decrease in myeloma cell apoptosis was also observed in myeloma-bearing MMP-7^−/−^mice as compared with WT mice (Fig. 1d). Accompanying the increase in tumor burden, myeloma-bearing MMP-7^−/−^ mice developed a more severe osteolytic bone disease, with decreased trabecular bone volume (Fig. 2a) and an increase in osteolytic lesions (Fig. 2b) as compared with myeloma-bearing WT mice. Myeloma-bearing mice demonstrated a significant increase in osteoclast number and decrease in osteoblast number (Fig. 2c-d).

**Fig. 3:**
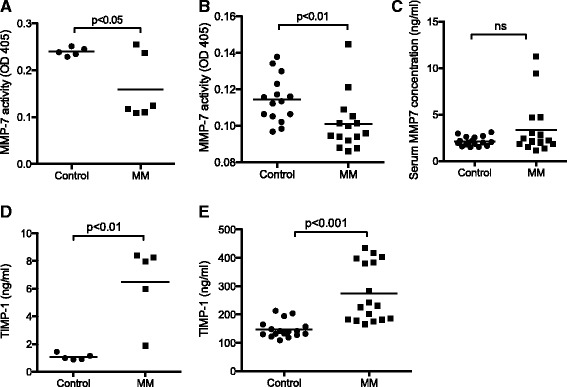
MMP7 activity is decreased in the serum of myeloma-bearing mice or patients with multiple myeloma. Analysis of MMP7 activity in the serum of myeloma-bearing mice (**a**) or control and myeloma patients (**b**) was detected using an MMP7 activity assay. **c** MMP7 concentration in serum of control and myeloma patients, as measured by ELISA. An increase in the serum concentration of TIMP-1 was detected by ELISA in myeloma-bearing mice (**d**) and patients with multiple myeloma (**e**). Data are presented as mean. Mice; control *n* = 5; MM *n* = 6. Patients; control, *n* = 16; MM, *n* = 16. ns = not significant. Statistical analysis by Mann Whitney *U* test

Further support for a role for MMP-7 in myeloma pathogenesis was provided by a significant reduction in MMP-7 activity in serum of myeloma-bearing mice as compared to non-tumor controls (Fig. 3a). The 5T model of myeloma has a number of well-characterized similarities with human myeloma. In order to determine whether the role of MMP-7 in murine myeloma pathogenesis translated to the clinical setting, we measured the concentration and activity of MMP-7 in newly diagnosed multiple myeloma patients. Patient samples were age-, sex- and BMI-matched to normal controls. A significant reduction in MMP-7 activity was detected following an MMP-7 substrate-based activity assay (Fig. 3b). In contrast to this reduction in activity levels, no difference was detected in the serum concentrations of MMP-7 (Fig. 3c).

Since MMP-7 activity is decreased in myeloma in vivo, both in patients with multiple myeloma and in a murine model of myeloma, we sought to determine whether the endogenous MMP inhibitors were elevated in these samples. Of the four TIMP family members, TIMP-1 is the most effective inhibitor of MMP-7 [22]. RT-PCR analysis revealed that 5TGM1 cells and osteoblasts express TIMP-1 and we observed significantly higher levels of TIMP-1 in serum derived from multiple myeloma bearing animals compared to control (Fig. 3d). In keeping with this observation, we also found that serum from human multiple myeloma patients contained significantly higher levels of TIMP-1 compared to healthy controls (Fig. 3e).

**Fig. 4:**
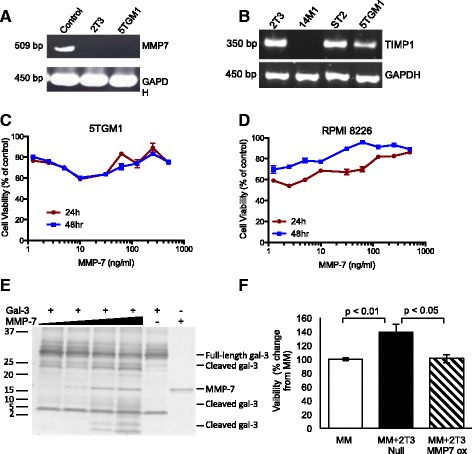
MMP-7 has limited effects on MMP-7 viability. **a** MMP-7 mRNA was measured in 2T3 osteoblasts and 5TGM1 myeloma cells. **b** TIMP-1 mRNA was measured in 2T3 osteoblasts, 14M1 myeloma-associated bone marrow stromal cells, ST2 bone marrow stromal cells and 5TGM1 myeloma cells. Treatment of 5TGM1 (**c**) or RPMI 8226 (**d**) myeloma cells with recombinant MMP-7 had limited effects on cell viability (*n* = 3). **e** Recombinant MMP7 can cleave murine galectin-3 into distinct fragments. 100 ng galectin-3 was incubated with increasing concentrations of MMP-7 (10–100 ng) and cleavage fragments detected by silver staining. **f** Over-expression of MMP-7 in 2 T3 osteoblasts decreased myeloma cell viability in a coculture of myeloma cells and osteoblasts (*n* = 3). Data represent mean ± SEM. Statistical analysis by one-way ANOVA and Tukey-Kramer posthoc test

The significant increase in both tumor burden and the associated osteolytic bone disease in MMP-7^−/−^ mice suggest that host-derived MMP-7 plays a specific tumor-suppressive role in myeloma. Myeloma cells were not found to express MMP-7, but instead expressed high concentrations of the endogenous MMP inhibitor TIMP-1 (Fig. 4a-b). Treatment of murine and human myeloma cells with increasing concentrations of recombinant MMP-7 in serum-free media had a limited effect to reduce cell viability, that was not dose-dependent (Fig. 4c-d). Similarly, flow cytometric analysis of apoptosis revealed no significant increase in apoptosis or necrosis following treatment with MMP-7, and western blot analysis confirmed no significant increase in cleaved caspase-3 or cleaved PARP (data not shown). Confirmation of functional MMP-7 activity was demonstrated by cleavage of the substrate galectin-3 (Fig. 4e). To begin to determine whether the tumour suppressive effect of MMP7 may be mediated via cells of the host microenvironment, myeloma cells were cultured in the presence of 2T3 preosteoblasts. This resulted in an increase in myeloma cell viability that was prevented by overexpression of MMP-7 in preosteoblasts (Fig. 4f).

## Conclusions

The present study identifies an unexpected role for MMP-7 in myeloma pathogenesis, with a striking increase in myeloma tumor burden and osteolytic bone disease in myeloma-bearing MMP-7 deficient mice, as compared to wild-type controls. These in vivo murine myeloma studies are supported by clinical evidence demonstrating a significant reduction in MMP-7 activity in patients with multiple myeloma. These studies demonstrate that host-derived MMP-7 plays a suppressive role in myeloma pathogenesis.

The increase in myeloma tumor burden in response to a loss of host-derived MMP-7 is in distinct contrast to previous studies in prostate cancer and breast cancer osteolysis, where tumor burden and osteolytic bone disease were decreased in MMP-7 deficient mice [2, 3]. MMP-7 is highly expressed by osteoclasts, and capable of cleaving RANKL to a soluble active form [2]. In murine models of prostate and breast cancer osteolysis, MMP-7 deficiency was associated with a reduction in the cleavage of RANKL to a soluble form and a decrease in osteoclasts at the tumor:bone interface [2, 3]. However, in the present study, no significant difference in RANKL was detected in myeloma-bearing MMP-7 deficient mice as compared to wild-type controls (data not shown). Notably, RANKL has been implicated in the pathogenesis of myeloma bone disease, in addition to that of breast and prostate cancer, although anti-RANKL is currently only approved for the treatment of solid tumor metastases [8]. The present study suggests a distinct difference in the response of myeloma to host-derived MMP-7, as compared to breast and prostate cancer. Although there are many similarities in cellular and molecular mechanisms of tumor growth and bone disease between myeloma and solid tumor bone metastases, there are also numerous examples of differences, including the relative contributions of PTHrP and Dkk1 [23–27]. However, there are limited examples of such opposing responses observed between the tumor types, highlighting the importance of studying MMP function in specific disease contexts.

To date, myeloma cells have been reported to express a number of MMPs, which are postulated to promote numerous aspects of tumor growth and osteolytic bone disease. MMP-2 and MMP-9 are the most widely studied and reported, with evidence for key roles in angiogenesis, tumor growth and extracellular matrix remodeling [10, 11, 14–16]. The contributions of host-derived MMPs to disease pathogenesis are poorly studied, due largely to difficulties in studying the host microenvironment in vivo. We have previously shown that myeloma tumor burden is decreased in MMP-9 deficient mice, supporting a key role for host-derived MMP-9 in myeloma pathogenesis [18]. In contrast, the role of MMP-7 is relatively unknown in myeloma, and is currently limited to the expression of MMP-7 by human myeloma cells, with resultant in vitro activation of MMP-2 [17]. In the present study, we have identified host-derived MMP-7 as having a tumor-suppressive role in myeloma. While the majority of MMPs promote tumor progression, inhibitory roles for MMPs have also been reported, including that of MMP-8 in oral cancer and lymph node metastasis [28–30]. In support of disease specific effects of MMP-s, MMP-3 is known to promote breast cancer progression but protect against the progression of squamous cell carcinoma [31–33]. As shown here, MMP-7 activity was reduced in the serum of both myeloma-bearing mice and in patients with multiple myeloma. Of note, no significant difference in MMP-7 concentration was detected, demonstrating the importance of studying enzymatic activity when investigating MMP regulation. An increase in the endogenous MMP inhibitor TIMP-1 was detected in patients with multiple myeloma, which may account for the reduction in MMP-7 activity, although it is likely that other mechanisms also contribute.

Genetic ablation of individual MMPs can result in the enhanced expression of other MMP family members with overlapping substrate specificity [34]. For example, previous reports with MMP-7 deficient mice have identified increased expression MMP-2 [35]. This indicates the potential for compensatory mechanisms in regards to the cleavage of substrates by other MMPs in our system [36]. However, the data suggests that the increased expression of MMPs is insufficient to rescue the effects of MMP-7 implying that the spatial and temporal expression of individual MMPs is important. We have previously shown for example that MMP-7 and MMP-9 expression from osteoclasts have distinct roles in the context of bone metastatic breast cancer [3].

MMP-7 has a number of reported substrates, including some associated with myeloma cell apoptosis such as FASL [37, 38]. However, cleavage of these substrates would be predicted to decrease their anti-tumor effect, rendering them unlikely candidates to explain the anti-myeloma effect of MMP-7 in the present study. Myeloma cells were not found to express MMP-7, and had a limited, inconsistent response to treatment with recombinant MMP-7 in vitro. This suggests that the myeloma-suppressive effect of MMP-7 is not merely a direct anti-tumour effect, but likely a complex mechanism involving cleavage of multiple substrates within the tumour-bone microenvironment. In support of this, the ability of preosteoblasts to promote myeloma cell viability was reduced in the presence of excess MMP-7 expressed by the host cells, highlighting the tumour-suppressive effect of MMP-7 in the myeloma-bone microenvironment and suggesting that the effect may be mediated at least in part by a substrate expressed by osteoblasts.

Despite many recent therapeutic advances, myeloma remains a fatal malignancy, in part due to its symbiotic relationship with the bone marrow microenvironment. Therefore, it is imperative to identify both tumor-derived and host-derived factors that contribute to disease pathogenesis. MMP-7 represents one such factor that is highly expressed within this specialized niche. Our studies have collectively identified an unexpected role for MMP-7 in myeloma pathogenesis, whereby a loss of MMP-7 promotes tumor growth and myeloma bone disease. MMPs represent attractive therapeutic targets for the treatment of many malignancies. However the failure of initial clinical trials using broad-spectrum MMP inhibitors reveals the complexity of MMPs as targets. The current study clearly demonstrates that inhibition of MMP-7 is an inappropriate approach for the treatment of myeloma and its associated bone disease. This is in direct contrast to breast and prostate cancer bone metastases, and highlights the necessity to elucidate the differential roles of individual MMPs in disease specific contexts.

## Abbreviations

DMEM: Dulbecco’s modified eagle medium
FAS-L: Fas ligand
MMP: Matrix metalloproteinase
RAG-2: Recombinase Activating Gene-2
RT-PCR: Reverse transcription polymerase chain reaction
TIMP-1: Tissue inhibitor of metalloproteinases-1
WT: Wild-type
